# Blimp-1-Mediated Pathway Promotes Type I IFN Production in Plasmacytoid Dendritic Cells by Targeting to Interleukin-1 Receptor-Associated Kinase M

**DOI:** 10.3389/fimmu.2018.01828

**Published:** 2018-08-07

**Authors:** Yi-An Ko, Yueh-Hsuan Chan, Chin-Hsiu Liu, Jian-Jong Liang, Tsung-Hsien Chuang, Yi-Ping Hsueh, Yi-Ling Lin, Kuo-I Lin

**Affiliations:** ^1^Genomics Research Center, Academia Sinica, Taipei, Taiwan; ^2^Institute of Microbiology and Immunology, National Yang-Ming University, Taipei, Taiwan; ^3^Program in Translational Medicine, Kaohsiung Medical University and Academia Sinica, Division of Allergy, Immunology and Rheumatology, Taipei Tzu Chi Hospital, Buddhist Tzu Chi Medical Foundation, New Taipei City, Taiwan; ^4^Institute of Biomedical Sciences, Academia Sinica, Taipei, Taiwan; ^5^Immunology Research Center, National Health Research Institutes, Miaoli, Taiwan; ^6^Institute of Molecular Biology, Academia Sinica, Taipei, Taiwan

**Keywords:** plasmacytoid dendritic cell, type I interferon, Blimp-1, interleukin-1 receptor-associated kinase M, antiviral response

## Abstract

Plasmacytoid dendritic cells (pDCs) are a specialized subset of DCs capable of rapidly producing copious amounts of type I IFN (IFN-I) in response to viral infections. The mechanism regulating rapid production of IFN-I after pDCs are exposed to viral nucleic acids remains elusive. Here, we show that the transcription factor Blimp-1 is promptly induced in pDCs after exposure to TLR7 and TLR9 ligands *via* a unique Ras-related C3 botulinum toxin substrate (Rac)-mediated pathway. Deletion of the *Prdm1* gene encoding Blimp-1 impaired production of IFN-I, but not other cytokines, upon viral infection or treatment with CpG DNA in pDCs. Accordingly, mice lacking Blimp-1 in DCs failed to produce IFN-I after CpG stimulation and did not mount proper antiviral responses following flavivirus infection. The development of pDCs in bone marrow as well as the induction of several activation markers, such as CD86, CD69, and MHCII, by CpG stimulation was generally not affected by the absence of Blimp-1. Mechanistically, we found that Blimp-1 controls the activation of IKKα and IRF7 by directly suppressing *interleukin-1 receptor-associated kinase 3* (*Irak3*), a negative regulator of TLR signaling, in pDCs. Together, we identify a Blimp-1-dependent pathway that rapidly facilitates IFN-I production by relieving interleukin-1 receptor-associated kinase M, encoded by *Irak3*, in pDCs.

## Introduction

Plasmacytoid dendritic cells (pDCs) are a distinctive subset of DCs with low abundance and a short lifespan (1). They produce copious amounts of type I IFN (IFN-I) by utilizing highly expressed TLR7 and TLR9 to sense pathogen-derived single-stranded RNA and unmethylated DNA, respectively (2–4). Besides IFN-I, pDCs also secret proinflammatory cytokines to combat early phase infection, including IL-6, IL-12, and TNF-α. These responses are accompanied by the upregulation of MHCII and co-stimulatory molecules that allow bridging activation of adaptive immunity (5). Aberrant pDC-derived IFN-I production is associated with the activation and expansion of auto-reactive T and B cells in autoimmune diseases (6). However, despite the importance of pDCs in the antiviral response and autoimmunity, the underlying regulatory pathways that contribute to the rapid large-scale production of IFN-I remain elusive.

Blimp-1, a transcription factor, is critical for regulating differentiation of mature B cells into plasma cells (7). It also plays important roles in several other immune cell lineages. For example, Blimp-1 negatively regulates the homeostasis of CD8^−^ conventional DCs (cDCs) and is essential for cDC maturation in response to stimulation (8). In particular, Blimp-1 participates in the regulation of the tolerogenic function of DCs. DC-specific deletion of *Prdm1*, the gene encoding Blimp-1, results in a lupus-like syndrome in female mice that is characterized by elevated serum autoantibodies, enhanced germinal center formation, and increased follicular T helper cells (9). However, whether Blimp-1 plays a functional role in pDCs remains unknown. Given that TLR ligands can induce Blimp-1 in several immune cell lineages (10), we here would like to investigate whether Blimp-1 is involved in the regulation of IFN-I production in pDCs.

## Materials and Methods

### Mice

*Prdm1^f/f^* mice (11) were crossed with CD11c-Cre or R26CreER mice, both purchased from The Jackson Laboratory, to generate *Prdm1^f/f^*CD11c-Cre^+/−^ (CKO-11c), *Prdm1^f/f^*ER-Cre^+/−^ (CKO-ER), and their littermate control *Prdm1^f/f^*CD11c-Cre^−/−^ (Ctrl-11c) or *Prdm1^f/f^*ER-Cre^−/−^ (Ctrl-ER) mice. To avoid the autoimmune phenotypes of female CKO-11c mice (9), only male CKO-11c and male littermate control mice were used in all experiments. *Tlr7* knockout (KO) (12) and Blimp-1-yellow fluorescent protein (YFP) reporter mice (13) were purchased from The Jackson Laboratory, and *Tlr9* KO (obtained from Dr. Shizuo Akira) (14) mice were paired with wild-type C57BL/6 mice (purchased from the National Laboratory Animal Center, Taipei, Taiwan). All mice were housed and bred in the specific pathogen free conditions in the animal facility of Institute of Cellular and Organismic biology at Academia Sinica. Animal experimental protocols were approved by IACUC of Academia Sinica.

### Reagents

Type-A CpG oligonucleotides (ODN2216), type-C CpG oligonucleotides (ODN2395), Imiquimod (R837), and poly(I:C) were purchased from InvivoGene. The lipopolysaccharide (*E. coli*. O26:B6) was obtained from Sigma-Aldrich Co. For virus infection, influenza A virus (H1N1/WSN, from Dr. Jia-Tsrong Jan), herpes simplex virus-1 (KOS strain, from Dr. Chia-Chi Ku), respiratory syncytial virus (A2 strain, from Dr. Joe Yen-Hung Chow), and Japanese encephalitis virus (JEV RP-9 strain, from Dr. Yi-Ling Lin) were used. In some experiments, the FLpDCs were pretreated with Rac inhibitor, EHop-016 (Calbiochem) for 1 h, followed by CpG-A or R837 stimulation.

### *In Vivo* Challenge and Plaque Assay

A neurovirulent JEV strain, RP-9, was used for the induction of encephalitis in CKO-11c and Ctrl-11c mice following the procedures described previously (15). Briefly, mice were anesthetized and intracerebrally injected with 10 µl of PBS to damage the brain–blood barrier followed by intraperitoneal inoculation with 5 × 10^4^ PFU of RP-9 virus. Sera were collected at indicated time points after infection and the mice were observed daily for 14 days to record lethality. Anti-PDCA-1 antibody (BX444; BioXcell) and rat IgG1 isotype control (HRPN; BioXcell) antibody were used to test the significance of pDCs in JEV infection *in vivo* and were injected three times (250 μg/injection) at 24-h intervals before infection.

For *in vivo* CpG-A challenge, 5 µg ODN2216 was mixed with 30 µl DOTAP, the liposomal transfection reagent, and incubated at room temperature for 15 min. Mice were intravenously injected with CpG-A plus DOTAP, or DOTAP alone. After 6 h, IFN-α and cytokines in sera were determined as previously described (16, 17).

To quantify JEV virus amounts, whole brain homogenates were harvested from Ctrl-11c and CKO-11c mice 6 days after JEV infection. BHK-21 cells were used for plaque assays as described previously (15). Briefly, brain homogenates were serially diluted and added into 80% confluent BHK-21 cells. After 2 h, the supernatant was removed and the infected BHK-21 cells were overlaid with 1% agarose-RPMI solution (SealPlaque, FMC BioProducts), followed by incubation at 37°C. Four days later, cells were fixed and stained with 0.5% crystal violet, and then the plaque numbers were counted.

### Cell Preparation, Stimulation, and Transfection

Splenic CD11c^+^ DCs were enriched by using positive selection with mouse CD11c microbeads (Miltenyl Biotec), the CD11c^int^B220^+^Siglec-H^+^ pDCs, or CD11c^high^B220^−^Siglec-H^−^ cDCs were sorted by cell sorter and cultured in RPMI 1640 supplemented with 10% FBS, 50 µM 2-ME, 100 U/ml penicillin, and 100 µg/ml streptomycin. FLpDCs were generated as previously described (18). Bone marrow (BM) cells were harvested from the femurs and tibiae of mice. Red blood cells were lyzed and single cell suspensions were cultured in RPMI 1640 supplemented with 50 ng Flt3 ligand (PeproTech) at a density of 1 × 10^6^ cells/ml for 9 days. To delete *Prdm1* allele *in vitro*, BM cultures from CKO-ER and Ctrl-ER mice were supplied with 500 nM 4-hydroxytamoxifen (4-OHT, Sigma-Aldrich). Nine days later, CD11c^+^ cells that were at least 90% confluent were used to enrich CD11c^+^B220^+^Bst2^+^Siglec-H^+^ pDCs after B220 microbeads isolation (Miltenyl Biotec). The purified pDCs were stimulated with 1 µM CpG-A (InvivoGen), CpG-C (InvivoGen), or 2 µg/ml R837 (InvivoGen). cDCs were treated with 50 ng/ml poly(I:C) (InvivoGen) or 10 ng/ml LPS (Sigma-Aldrich) at a density of 1 × 10^6^ cells/ml for the indicated time points. For virus infection, pDCs (1 × 10^6^ cells/ml) were infected with influenza H1N1 (WSN strain) at a titer of 1 × 10^4^ TCID50/ml. Herpes simplex virus 1 (HSV-1) (KOS strain) and respiratory syncytial virus (RSV A2 strain) were applied at an MOI of 1 and JEV (RP9 strain) was used at an MOI of 10 for 24 h.

Human peripheral blood mononuclear cells (PBMCs) from healthy donors were isolated by density gradient centrifugation with Ficoll-Paque at 400 × *g* for 30 min without brake at 22°C. The mononuclear cells were carefully isolated from the interphase and the BDCA2^+^ pDCs were further purified by plasmacytoid dendritic cell isolation kit II (Miltenyl Biotec). In some experiments, the purified pDCs were stimulated with 1 µM CpG-A or influenza H1N1 (WSN strain) at a titer of 10^4^ TCID50/ml for 24 h. Blood samples were from Taipei Blood Center. The consent procedures of collection of samples from healthy donors were approved by the Academia Sinica Research Ethics Committee.

To knock down interleukin-1 receptor-associated kinase M (IRAK-M) expression, the FLpDCs generated from Ctrl-ER or CKO-ER mice were isolated and transfected with small-interfering RNA (siRNA) against *interleukin-1 receptor-associated kinase 3* (*Irak3*) or the control siRNA by TurboFect (Thermo Scientific). The transfection procedure was performed as previously described (19). Briefly, 1.5 µg siRNA were diluted in 50 µl serum-free RPMI1640 containing 1 µl TurboFect for 15 min at room temperature. After incubation, the mixtures were added to FLpDCs in a final volume of 550 µl. The target sense sequences were synthesized by TOOLS Biotechnology Co. The Irak3 siRNA sequences are #1:5′-GGGAAGACUUUCCGUUAAATT-3′, #2:5′-GGCUGGAUGUUCGUCAUAUTT-3′, and #3:5′-GCAGAGUUCUACCAUAAAUTT-3′, and the FAM tagged control sequences are 5′-UUCUCCGAACGUGUCACGUTT-3′.

### RNA Isolation and RT-Quantitative PCR (RT-qPCR)

Total RNAs were extracted by Isol-RNA Lysis Reagent (5 PRIME), and subjected to reverse transcription by High-Capacity cDNA Reverse Transcription Kits (Applied Biosystems). Gene specific primer sets were used to perform the qPCR analysis by using Applied Biosystems StepOne™ Real-Time PCR System. Taqman probe sets including mouse *Prdm1* (Mm 01187285_m1) and human *PRDM1* (Hs 00153357_m1) were purchased from Applied Biosystems. The specific primer sequences for SYBR green detection are listed below: *Ifna4*, 5′-GCAATGACCTCCATCAGCAGCT-3′, and 5′-GTGGAAGTATGTCCTCACAGCC-3′; *Ifna5*, 5′-GGATGTGACCTTCCTCAGACTC-3′, and 5′-CACCTTCTCCTGTGGGAATCCA-3′; *Ifnb1*, 5′-GCCTTTGCCATCCAAGAGATGC-3′, and 5′-ACACTGTCTGCTGGTGGAGTTC-3′; *Il6*, 5′-ACAAGTCGGAGGCTTAATTACACAT-3′, and 5′-AATCAGAATTGCCATTGCACAA-3′; *Il12p40*, 5′-TTGAACTGGCGTTGGAAGCACG-3′, and 5′-CCACCTGTGAGTTCTTCAAAGGC-3′; *Tnfa*, 5′-GACCCTCACACTCAGATCATCTTCT-3′, and 5′-CCTCCACTTGGTGGTTTGCT-3′; *Irak3*, 5′-CTGCAAAGTGGTGCTGGATGAC-3′, and 5′-GCTTTGCAGAGAAGTTCCGAGG-3′; *Tcf4*, 5′-CCTCCAATCCTTCAACTCCTGTG-3′, and 5′-TCCAAACGGTCTTCGATTCGGC-3′; *Ikzf1*, 5′-CCACCACGAGATGGCAGAAGAC-3′, and 5′-GGCATGTCTGACAGGCACTTGT-3′; *Irf8*, 5′-CAATCAGGAGGTGGATGCTTCC-3′, and 5′-GTTCAGAGCACAGCGTAACCTC-3′; *Tlr7*, 5′-GTGATGCTGTGTGGTTTGTCTGG-3′, and 5′-CCTTTGTGTGCTCCTGGACCTA-3′; *Tlr9*, 5′-GCTGTCAATGGCTCTCAGTTCC-3′, and 5′-CCTGCAACTGTGGTAGCTCACT-3′; *Actin*, 5′-CATTGCTGACAGGATGCAGAAGG-3′, and 5′-TGCTGGAAGGTGGACAGTGAGG-3′.

### Nuclear and Cytoplasmic Proteins Extraction and Immunoblotting

Cell cytoplasmic and nuclear extracts were obtained by using NE-PER nuclear and cytoplasmic extraction reagents according to the manufacturer’s protocols (ThermoFisher). Immunoblotting was performed as previously described (8). The blots were probed with anti-IRF7 antibody (EPR4718; abcam), anti-Lamin-B (M-20; Santa Cruz Biotechnology), anti-IKKα (Cell Signaling), anti-AKT (Cell Signaling), anti-Osteopontin (Abcam), anti-p65 (C-20; Santa Cruz Biotechnology), anti-P50 (Santa Cruz Biotechnology), anti-STAT1 (Cell Signaling), anti-IRAK-M (ProSci), and anti-Blimp-1 (Abcam). The activation of IRF7, IKKα/β, AKT, and STAT1 were detected by phospho-specific antibodies against pIRF7 (Ser471/472; D6M2I; Cell Signaling), pIKKα/β (Ser176/180; 16A6; Cell Signaling), pAKT (Ser473; D9E; Cell Signaling), and pSTAT1 (Tyr701; 58D6; Cell Signaling). Representative blots from at least two independent experiments were shown.

Rac1 activation was detected by Rac1 activation assay kit (Abcam). Briefly, the total cell lysates were harvested from stimulated FLpDCs and incubated with PAK1 PBD beads at 4°C for 1 h. Rac1-GTP precipitate and the total lysate controls were analyzed by western blot analysis. Rac1 was detected by a specific mouse monoclonal antibody.

### ELISA

The supernatant from stimulated pDC culture or the serum collected from the CpG-A injected or JEV infected mice was harvested and subjected to ELISA analysis to determine the levels of IFN-α (PBL Assay Science), IL-6, and TNF-α (eBioscience) following the manufacturer’s protocols. Finally, 2 N H_2_SO_4_ was added to stop the reaction and absorbance at 450 nm was measured using a microplate reader (SpectraMax M2).

### Flow Cytometry Analysis and Antibodies

Single cell suspensions were prepared for surface staining of the cells with fluorochrome-conjugated antibodies against Flt3 (A2F10), Bst2 (ebio927), and Siglec-H (ebio440c) were purchased from eBioscience, B220 (RA3-6B2), CD4 (RM4-5), CD8 (53-6.7), CD11b (M1/70), CD3 (145-2c11), and CD86 (GL1) were purchased from BD, CD69 (H1.2F3), CD19 (6D5), CD49b (Dx5), MHCII (M5/114.15.2), and Ly-6c (HK1.4) were purchased from BioLegend, and Ly49Q (2E6) were purchased from Medical & Biological Laboratories Co. After incubating on ice for 15 min, the cells were washed twice and analyzed by BD FACS canto II flow cytometer. In some experiments, mouse splenic pDCs and cDCs were sorted by BD FACS Aria II system.

### Chromatin Immunoprecipitation (ChIP) Assay

To detect Blimp-1 binding to the endogenous target sites, a ChIP assay was performed according to previously described procedures (20). Basically, 5 × 10^7^ Flt3L cultured pDCs from C57BL/6 mice were stimulated with 1 µM CpG-A for 4 h and fixed with 1% formaldehyde at 37°C for 15 min and quenched with 125 mM glycine. The sheared chromatins were incubated with goat anti-Blimp-1 antibody (Abcam) or goat IgG isotype antibody (Abcam) at 4°C overnight. The antibody-chromatin immunocomplexes were pulled down by the protein-G magnetic beads and eluted at 65°C for 30 min. Immuneprecipitated DNA was isolated and analyzed by real-time qPCR. The primer sequences used in qPCR are listed below: site 1, 5′-AGGAATCTTGGTGACAATTTGGC-3′, and 5′-GACGGTAAAAGCTAGGGTGCTCT-3′; site 2, 5′-CCAAAATGATGGACTGTGGCC-3′, and 5′-CCCTGATGAAAGCAGATTCGG-3′; site 3, 5′-GCAAAGTGGCCCGATTGAGAGTA-3′, and 5′-CGGCCTTCAAAACAAAATGTTCTG-3′; site 4, 5′-TGTTGTTCTTCCTATGGGGTTGC-3′, and 5′-AACCATTGGACTGAGCACAGGGT-3′; site 5, 5′-TCTGAGTTTGACGCCCCAGTACA-3′, and 5′-TGCGCAAGTGCACATGTACATGA-3′; and *Gapdh*, 5′-GGGTTCCTATAAATACGGACTGC-3′, and 5′-CTGGCACTGCACAAGAAGA-3′.

### Statistical Analysis

Statistical significance was determined by using the two-tailed unpaired Student’s *t*-test. Data represent mean ± SEM. The differences in mouse survival between two groups were analyzed by log-rank (Mantel-Cox) test. Results from independent biological replicates were used in statistical analysis. *p < 0.05; **p < 0.01; ***p < 0.001.

## Results

### Blimp-1 Is Induced After TLR7/9 Stimulation in pDCs

**Figure 1:**
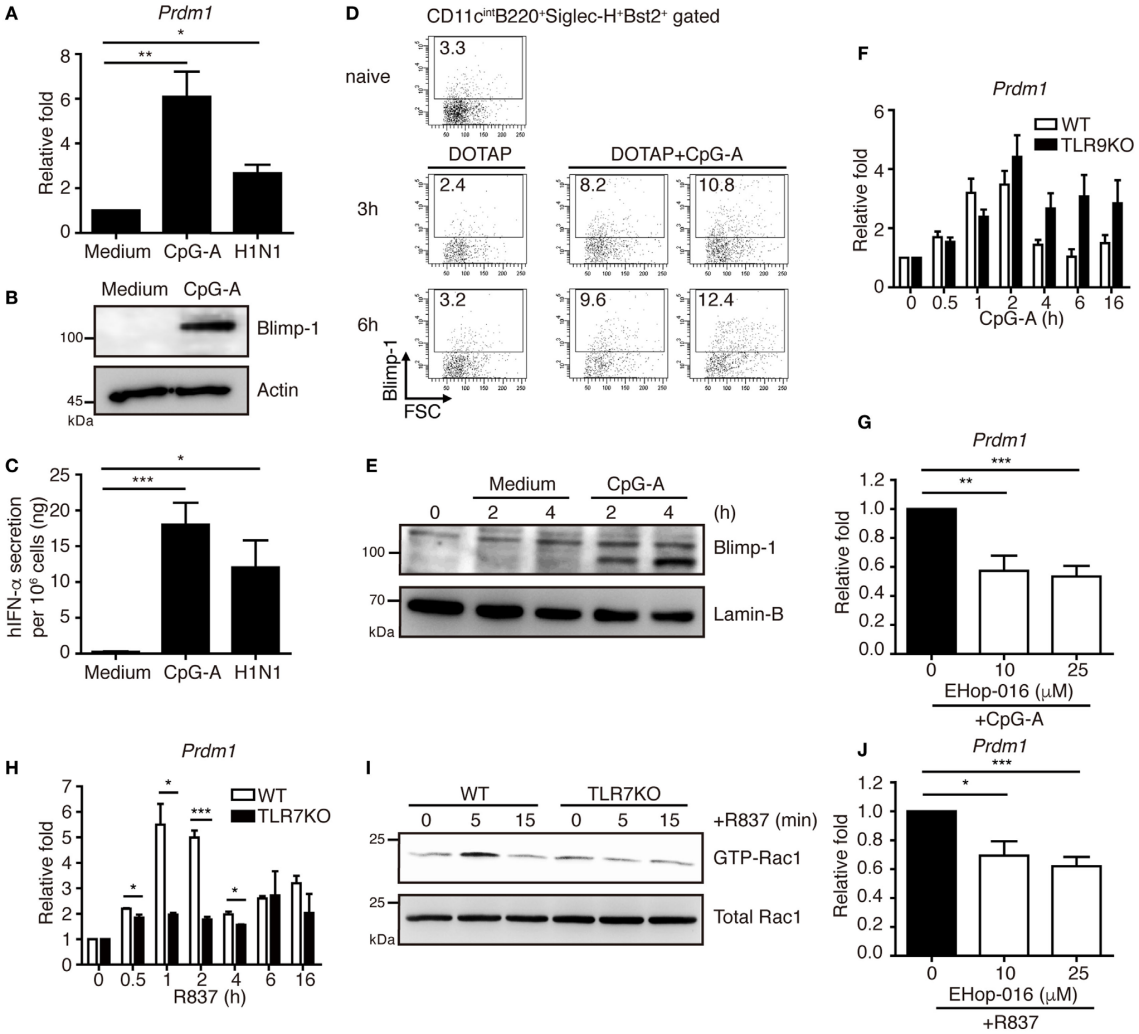
Blimp-1 was induced *via* Rac activation after TLR7/9 ligand treatment in plasmacytoid dendritic cells (pDCs). **(A)** RT-quantitative PCR (RT-qPCR) showing Blimp-1 mRNA in human pDCs 24 h after treatment with medium alone, 1 µM CpG-A and H1N1 at a titer of 10^4^ TCID50/ml. **(B)** Blimp-1 protein levels were determined by immunoblotting in human pDCs 24 h after treatment with medium alone or 1 µM CpG-A. **(C)** ELISA showing the levels of IFN-α produced by human pDCs as described in **(A)**. **(D)** Blimp-1-yellow fluorescent protein reporter mice were intravenously injected with DOTAP alone or DOTAP + CpG-A. The frequency of Blimp-1^+^ pDCs in splenic CD11c^int^B220^+^Siglec-H^+^Bst2^+^ gate was examined at indicated time after infection. The frequency of Blimp-1^+^ pDCs from untreated group (naïve) was shown for comparison. **(E)** Nuclear Blimp-1 protein levels were detected by immunoblotting in mouse FLpDCs stimulated with medium alone or 1 µM CpG-A at indicated time points. Freshly isolated FLpDCs at 0 h, before addition of medium alone or CpG-A, were also used as the control. **(F)** RT-qPCR showing the Blimp-1 mRNA levels in *Tlr9* knockout (KO) FLpDCs treated with 1 µM CpG-A. **(G)** RT-qPCR showing Blimp-1 mRNA levels in FLpDCs after 1 h pre-treatment with EHop-016 and further treatment with 1 µM CpG-A for 1 h. **(H)** RT-qPCR showing the Blimp-1 mRNA levels in *Tlr7* KO FLpDCs treated with 2 µg/ml R837 for 1 h. **(I)** Rac1 activation determined by PAK1 PBD agarose beads pulled down and immunoblotting with antibody against Rac1 in FLpDCs from WT and TLR7 KO mice after stimulation with 2 µg/ml R837. **(J)** RT-qPCR showing Blimp-1 mRNA expression in FLpDCs after 1 h pre-treatment with EHop-016 and further treatment with 2 µg/ml R837 for 1 h. Data represent the mean ± SEM and were analyzed by two-tailed unpaired Student’s *t*-test [*n* = 3−6 in **(A)**, 4−7 in **(C)**, 3 in **(F)**, 5−6 in **(G)**, 3 in **(H)**, and 4−5 in **(J)**]. *p < 0.05; **p < 0.01; ***p < 0.001.

We first examined the expression of Blimp-1 in pDCs after stimulation. Human PBMCs were isolated from healthy donors and the BDCA2^+^ pDCs were purified. Compared with the pDCs treated with medium alone, Blimp-1 expression in pDCs was upregulated after treatment with CpG-A or influenza virus (H1N1) infection (Figures 1A,B), which induced high IFN-I production (Figure 1C). We then examined if Blimp-1 is expressed in mouse pDCs, characterized as CD11c^int^B220^+^Siglec-H^+^Bst2^+^ (21–23) (Figure S1 in Supplementary Material). The Blimp-1-YFP reporter mice that express YFP under the control of Blimp-1 regulatory element (13) were used to track the expression of Blimp-1. Similar to human pDCs, a rapid induction of Blimp-1 in mouse splenic pDCs was detected 3 h after intravenous injection of DOTAP/CpG-A, as compared with the DOTAP injected group (Figure 1D). This rapid induction of Blimp-1 was also observed after exposure of Flt3-ligand-cultured bone marrow (BM)-derived pDCs (FLpDCs) to the CpG-A as compared with the medium treated FLpDCs (Figure 1E). However, to our surprise, a lack of TLR9 did not affect Blimp-1 expression (Figure 1F). Given that TLR7 and TLR9 are endosomal receptors, and that TLR ligands transiently stimulate endocytosis in DCs (24), we suspected the induction of Blimp-1 in FLpDCs may occur upstream of TLR activation. Rac-1, a small G protein, is activated by stimulation with TLR9 ligand; however, this occurs independently of TLR9 activation (25). Indeed, the induction of Blimp-1 in FLpDCs was significantly reduced following the treatment with EHop-016, a Rac inhibitor that docks at the guanine nucleotide exchange factor (GEF) binding pocket of Rac to inhibit Rac activation (26) (Figure 1G). In addition, Blimp-1 can be induced by R837, the TLR7 ligand, in FLpDCs, but the induction of Blimp-1 was diminished when TLR7 is deficient (Figure 1H). This finding was correlated with the defective Rac-1 activation after R837 treatment in TLR7 KO FLpDCs (Figure 1I). Moreover, inhibition of Rac activity also decreased Blimp-1 induction after R837 treatment in FLpDCs (Figure 1J). These combined data suggest that Blimp-1 induction in pDCs is mediated by Rac activation soon after exposure to TLR7 and TLR9 ligands.

### Blimp-1 Is Essential for IFN-I Production in pDCs

**Figure 2:**
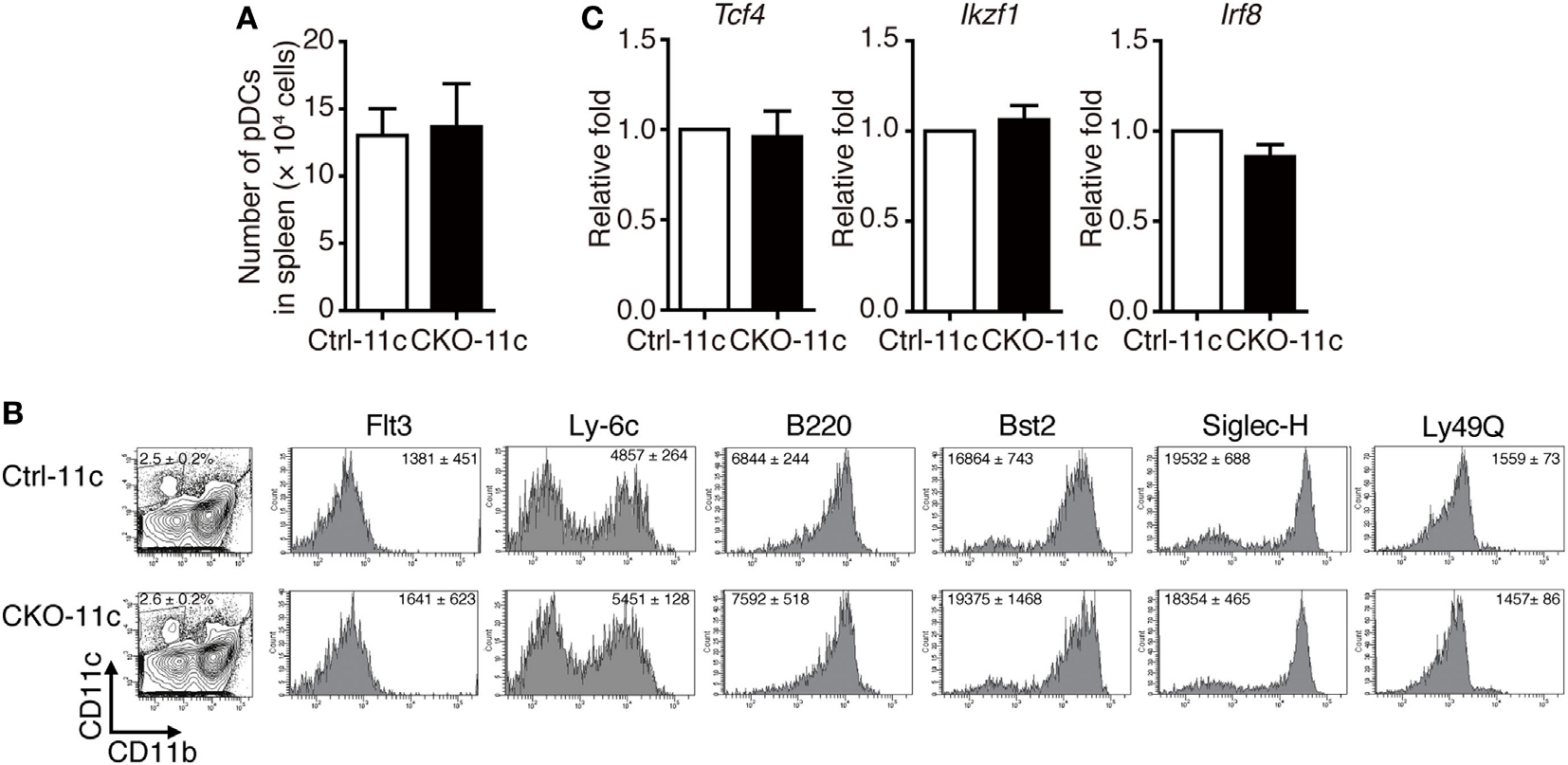
Comparable plasmacytoid dendritic cell (pDC) development in CKO-11c mice and Ctrl-11c mice. **(A)** pDC numbers in the spleen of Ctrl-11c and CKO-11c mice were enumerated. **(B)** Flow cytometric analysis showing the expression of various pDC maturation markers in CD11b−CD11c+ bone marrow cells isolated from Ctrl-11c or CKO-11c mice. The frequency of the CD11b^−^CD11c^+^ population and the mean fluorescence intensity of the staining in each histogram are indicated. **(C)** RT-quantitative PCR showing the mRNA expression levels of E2-2 (*Tcf4*), Ikaros (*Ikzf1*), and IRF8 in splenic pDCs isolated from Ctrl-11c or CKO-11c mice. Results represent the mean ± SEM and were analyzed by two-tailed unpaired Student’s *t*-test [*n* = 3 in **(A−C)**].

To determine the functions of Blimp-1 in pDCs, we generated mice carrying a conditionally deleted *Prdm1* allele. *LoxP*-flanked *Prdm1* (*Prdm1^f/f^*) mice were crossed with mice expressing Cre recombinase under the control of the integrin alpha X (Itgax/CD11c) promoter, CD11c-cre, to obtain mice with a DC-specific *Prdm1* deletion, hereafter referred to as CKO-11c mice. Blimp-1 deletion efficiency was ascertained at both genomic DNA and mRNA levels in splenic pDCs and cDCs (Figures S2A,B in Supplementary Material), as well as in BM CD11c^+^ cells (Figures S2C,D in Supplementary Material). Because of the gender-specific autoimmune phenotype in female CKO-11c mice (9), only male mice were used in this study. First, we examined whether Blimp-1 regulated pDC development. The absolute counts of splenic pDCs from CKO-11c mice were similar to the littermate control, Ctrl-11c, mice (Figure 2A). According to the mouse model of sequential pDC development (23), reduced Blimp-1 in BM DC lineages did not appear to alter pDC development because the expression of various markers representing pDC developmental stages was comparable between BM pDCs in CKO-11c and Ctrl-11c mice (Figure 2B). Previous studies demonstrated that for pDCs to develop from progenitors in BM, several critical factors are required including Flt3, and the transcription factors E2-2 (encoded by *Tcf4*), Ikaros (encoded by *Ikzf3*), and IRF8 (23, 27). Cell surface Flt3 and the transcription factor mRNA levels were consistently equivalent in Ctrl-11c and CKO-11c pDCs (Figures 2B,C). Therefore, Blimp-1 may not be important for the development of pDCs.

**Figure 3:**
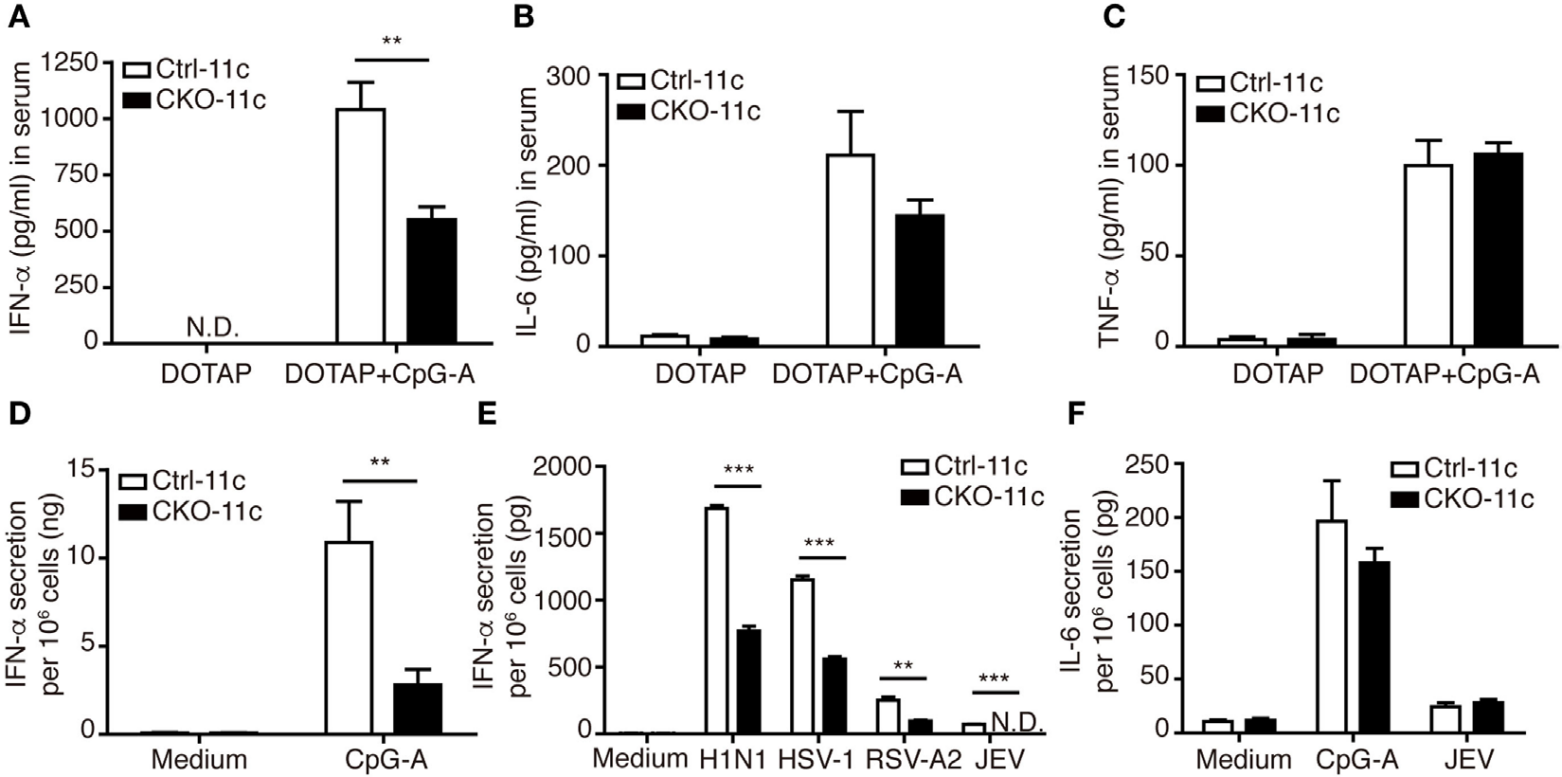
Type I IFN (IFN-I) production was impaired in CKO-11c mice and in plasmacytoid dendritic cells (pDCs) from CKO-11c mice. **(A)** ELISA showing the levels of IFN-α production in the serum of Ctrl-11c and CKO-11c mice 6 h after intravenous injection with 5 µg CpG-A + DOTAP or DOTAP alone. **(B,C)** ELISA determining the levels of IL-6 **(B)** and TNF-α **(C)** in serum from Ctrl-11c and CKO-11c mice from panel **(A)**. **(D,E)** ELISA determining the levels of IFN-α production at 24 h in medium alone treated, 1 µM CpG-A-stimulated or virus-infected splenic pDCs isolated from Ctrl-11c and CKO-11c mice. **(F)** ELISA measurement of the levels of IL-6 produced by Ctrl-11c and CKO-11c splenic pDCs at 24 h after treatment with medium alone, 1 µM CpG-A and Japanese encephalitis virus (JEV) at MOI of 10. Data represent the mean ± SEM and were analyzed by two-tailed unpaired Student’s *t*-test [*n* = 3 in DOTAP, 5−7 in CpG-A + DOTAP in **(A)**, 3−4 in **(B,C)**, 3 in medium, 6 in CpG-A treated group in **(D)**, 3 in **(E)**, and 3−4 in **(F)**]. **p < 0.01; ***p < 0.001. N.D. = not detectable.

Because the ability to produce large quantities of IFN-I is the hallmark of pDCs (28), we next examined whether Blimp-1 is involved in IFN-I production by pDCs. Blimp-1 was originally identified as binding to the positive regulatory domain I (PRDI) element of the *IFN-β* promoter and negatively regulating IFN-β expression (29). To our surprise, a significant reduction in IFN-α was detected in the sera of CpG-A/DOTAP treated CKO-11c mice compared with that of Ctrl-11c mice, while DOTAP injection did not induce IFN-α in both Ctrl-11c and CKO-11c mice (Figure 3A). By contrast, comparable amounts of proinflammatory cytokines IL-6 and TNF-α were detected in CpG-A treated Ctrl-11c and CKO-11c mice (Figures 3B,C). These results suggest a role for Blimp-1 in the control of IFN-I production. To verify whether intrinsic Blimp-1 expression in pDCs contributes to IFN-I production, splenic pDCs were isolated from Ctrl-11c and CKO-11c mice and stimulated with CpG-A and viruses including several single-stranded RNA viruses; influenza H1N1 virus, RSV-A2, and JEV, as well as a double-stranded DNA virus, HSV-1. Remarkably, IFN-α production by all stimuli was reduced in pDCs lacking Blimp-1 (Figures 3D,E). However, IL-6 production by pDCs was not affected in the absence of Blimp-1 (Figure 3F). Similar numbers of viable cells were found in control and Blimp-1-deficient splenic pDCs after treatment (Figures S2E in Supplementary Material). These data indicated that Blimp-1 plays a crucial role in antiviral responses in pDCs.

**Figure 4:**
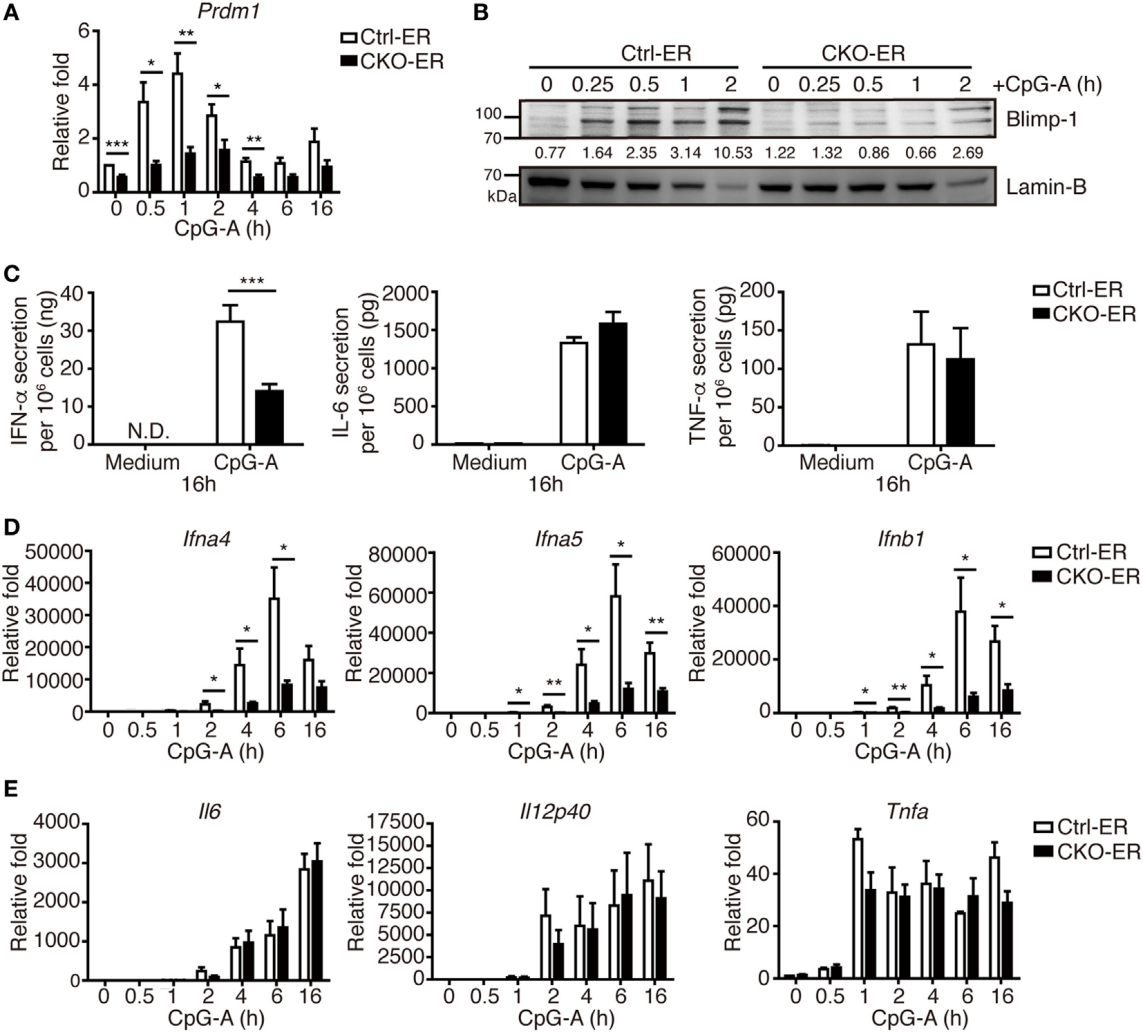
Inducible deletion of *Prdm1* confirmed the important role of Blimp-1 in type I IFN (IFN-I) production in plasmacytoid dendritic cells (pDCs). **(A,B)** RT-quantitative PCR (RT-qPCR) **(A)** and immunoblotting **(B)** showing Blimp-1 mRNA and protein levels in FLpDCs from Ctrl-ER and CKO-ER mice treated with 500 nM 4-OHT and then stimulated with 1 µM CpG-A at indicated time points. The quantitation of Blimp-1 in **(B)** was presented by the ratios of Blimp-1 band intensity vs. Lamin-B band intensity at each time point. **(C)** 4-OHT treated FLpDCs cultured from Ctrl-ER and CKO-ER mice were stimulated with 1 µM CpG-A or medium alone for 16 h, followed by ELISA to measure the levels of IFN-α, IL-6, and TNF-α production. **(D,E)** RT-qPCR showing mRNA levels of IFN-I **(D)** and proinflammatory cytokines **(E)** in 4-OHT treated FLpDCs from Ctrl-ER and CKO-ER mice after stimulation with 1 µM CpG-A at indicated time points. Data represent the mean ± SEM and were analyzed by two-tailed unpaired Student’s *t*-test [*n* = 6 in **(A)**, 3 in medium, 9−14 in CpG-A treatment in **(C)**, 7 in **(D)**, and 3−4 in **(E)**]. *p < 0.05; **p < 0.01; ***p < 0.001.

**Figure 5:**
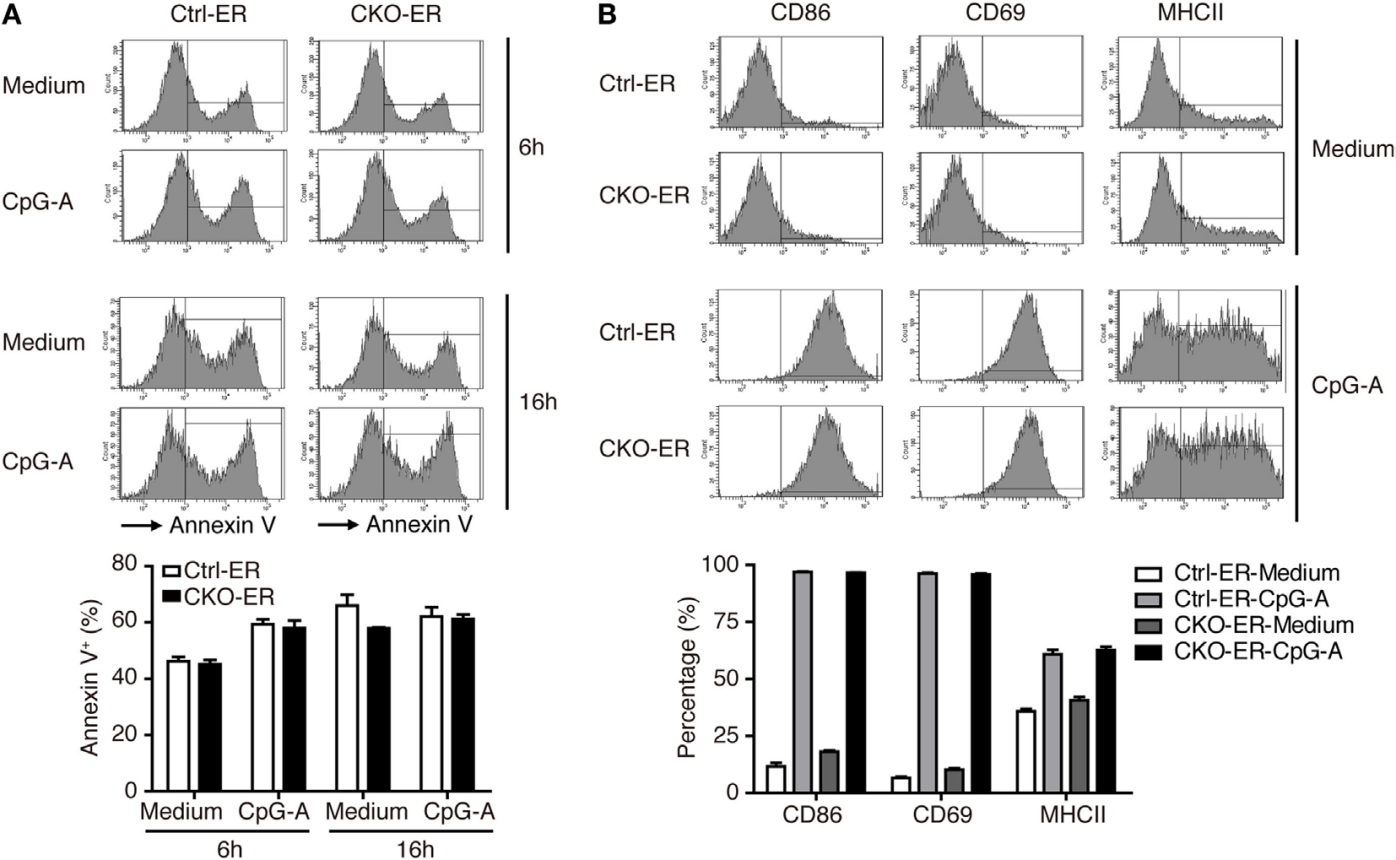
Activation and apoptosis were not affected in Blimp-1-deficient FLpDCs. **(A)** Flow cytometric analysis showing the frequency of Annexin V positive cells in 4-OHT treated Ctrl-ER or CKO-ER FLpDCs stimulated with 1 µM CpG-A or medium alone for 6 and 16 h. **(B)** Flow cytometric analysis of CD86, CD69, and MHCII expression on 4-OHT treated Ctrl-ER or CKO-ER FLpDCs with or without 16 h of 1 µM CpG-A stimulation. The positive frequency of each marker is indicated. Results represent the mean ± SEM and were analyzed by two-tailed unpaired Student’s *t*-test [*n* = 3 in **(A,B)**].

To exclude the possibility that this result might be caused by impaired pDC development that was not readily detected in our analysis, we crossed *Prdm1^f/f^* mice with mice carrying the inducible estrogen receptor/cre (ER-cre) in ubiquitous tissues (30). The resulting inducible *Prdm1* KO mice, termed CKO-ER mice, had almost 70% inducible deletion of *Prdm1* in FLpDCs after induction with 4-hydroxytamoxifen (4-OHT) (Figure S3A in Supplementary Material). Blimp-1 mRNA and protein levels were also significantly decreased in the 4-OHT treated FLpDCs from CKO-ER mice (Figures 4A,B). Of note, Blimp-1 protein expression was detected early at 15 min after CpG-A stimulation in 4-OHT treated FLpDCs from littermate controls, Ctrl-ER mice (Figure 4B). We verified that the deletion of *Prdm1 in vitro* during FL-mediated BM culture did not affect pDC development (Figures S3B,C in Supplementary Material). TLR7 and TLR9 expression was comparable between 4-OHT-treated FLpDCs derived from CKO-ER and Ctrl-ER mice (Figure S3D in Supplementary Material). We also ensured that 4-OHT had no obvious effects on Blimp-1 induction (Figure S3E in Supplementary Material). It is noted that we consistently showed defective IFN-I induction after CpG stimulation in 4-OHT-treated FLpDCs derived from CKO-ER mice compared with those from Ctrl-ER mice (Figures 4C,D); and there was no change in IL-6, IL-12p40, or TNF-α (Figures 4C,E). In addition to CpG-A, Blimp-1 mRNA levels were increased in FLpDCs after CpG-C treatment (Figure S3F in Supplementary Material). Consistently, IFN-α production was reduced in Blimp-1-deficient FLpDCs, while the production of IL-6 and TNF-α was comparable between control and Blimp-1-deficient FLpDCs after CpG-C treatment (Figure S3G in Supplementary Material). The reduced production of IFN-α in FLpDCs lacking Blimp-1 was not caused by enhanced cell death because the frequency of Annexin V-positive cells was similar in Blimp-1-deficient and control FLpDCs at 6 and 16 h after treatment (Figure 5A). Furthermore, Blimp-1-deficient FLpDCs appear to be activated in a similar manner to control FLpDCs, as evidenced by the comparable induction of CD86, CD69, and MHCII expression following CpG-A treatment (Figure 5B). Combined, these results show that Blimp-1 expression in pDCs selectively controls IFN-I production.

### Impaired Antiviral Responses in Blimp-1 Deficient-Mice

**Figure 6:**
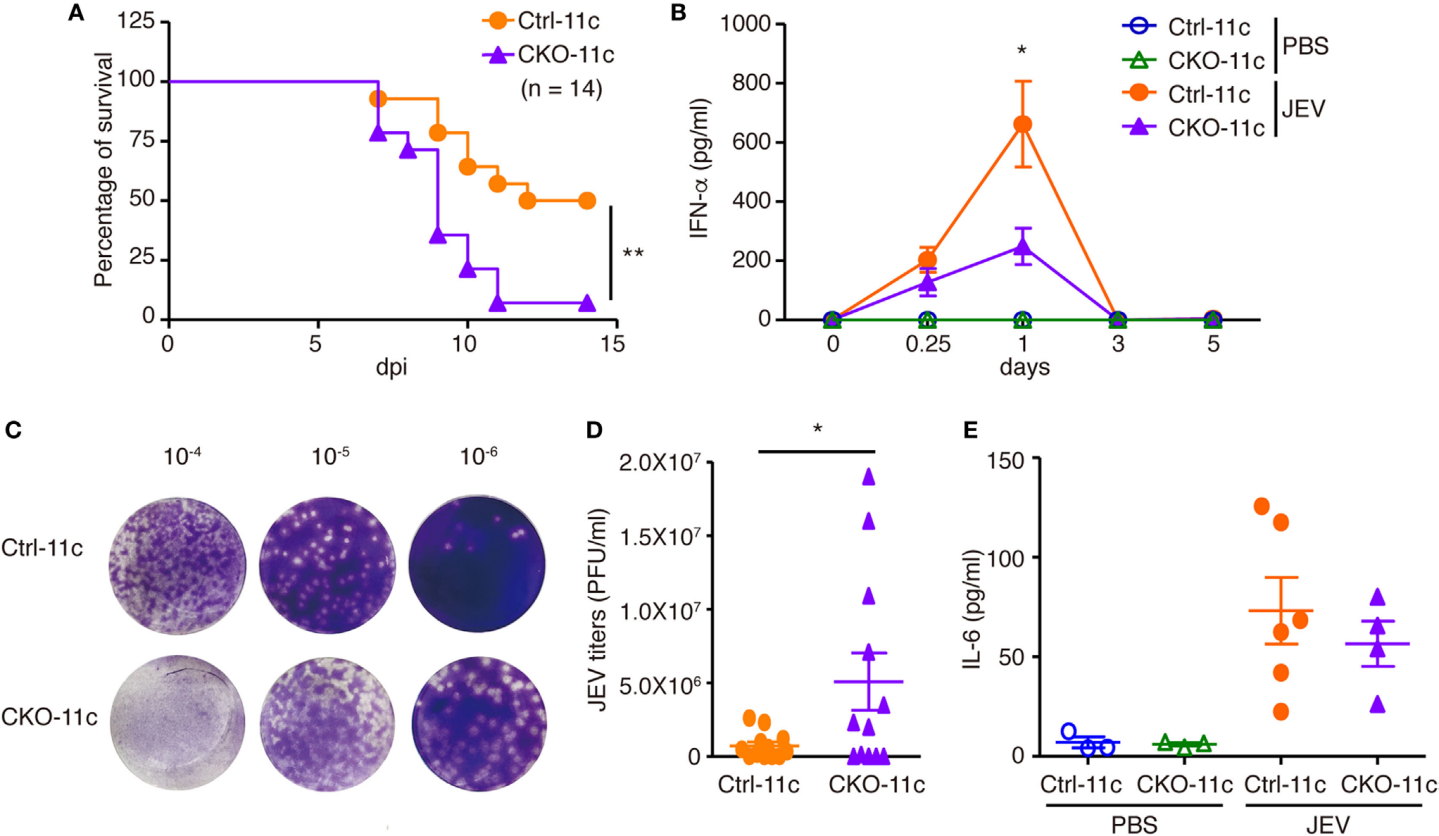
Impaired antiviral responses in mice lacking Blimp-1 in DCs. **(A)** Survival rates of Ctrl**-**11c or CKO**-**11c mice infected with Japanese encephalitis virus (JEV) (5 × 10^4^ pfu) by intraperitoneal (i.p.) injection followed by intracerebral (i.c.) needle injury. dpi: days post-infection. **(B)** ELISA measurement of IFN-α at different days post-PBS injection or post-JEV infection in Ctrl-11c and CKO-11c mice serum. **(C,D)** Plaque assay showing JEV titers in Ctrl**-**11c and CKO**-**11c mouse brains at 6 days post-infection **(C)**. Quantitation of results from panel **(C)** is **(D)**. **(E)** ELISA showing serum IL-6 levels 24 h after JEV infection or PBS injection in Ctrl-11c and CKO-11c mice. Data in **(A)** was analyzed by log-rank (Mantel-Cox) test (*n* = 14). Data in **(B,D,E)** represent the mean ± SEM and were analyzed by two-tailed unpaired Student’s *t*-test [*n* = 10−13 in 0.25 and 1 dpi and 3−5 in 0, 3, and 5 dpi and PBS injected group in **(B)**, 12 in **(D)**, and 3−6 in **(E)**]. *p < 0.05; **p < 0.01.

Type I IFN induction is essential for fighting viral infection, replication, and pathogenesis. Because pDCs with reduced Blimp-1 expression had defective IFN-I production after viral infection, we examined the importance of Blimp-1 in antiviral responses *in vivo*. The infection of JEV, a flavivirus, is highly sensitive to IFN-I production, but it is unaffected in mice lacking components of adaptive immunity (15, 31). Furthermore, our data showed that IFN-α production was reduced in Blimp-1-deficient pDC culture after JEV infection. Toward this end, we first examined the importance of pDCs in the clearance of JEV infection in mice. According to the reported procedures (32), which depleted the mouse pDCs *in vivo* but avoided the inadvertent depletion of other immune cells activated after virus infection, we injected mice with three shots of anti-PDCA-1 antibody or isotype control antibody at 24-h intervals only before JEV infection (Figure S4A in Supplementary Material). Administration of anti-PDCA-1 antibody with this strategy caused nearly complete depletion of pDCs in mouse spleen (Figures S4B−D in Supplementary Material), which was linked with increased mortality and reduced IFN-α production in sera after JEV infection (Figures S4E,F in Supplementary Material). The frequency of other immune cell types, including cDCs, myeloid cells, B cells, T cells, NK cells, sand NKT cells, at before or 3 days after JEV infection was not affected by anti-PDCA-1 antibody treatment (Figures S4G,H in Supplementary Material). To address further the roles of Blimp-1 in antiviral responses, Ctrl-11c and CKO-11c mice were intraperitoneally injected with JEV, followed by intracerebral damage of the brain–blood barrier. We found that CKO-11c mice were much more susceptible to JEV infection and have higher mortality rate (Figure 6A). This increased susceptibility was correlated with diminished serum IFN-α levels quickly after JEV infection (Figure 6B) and elevated viral titers in the brain (Figures 6C,D). However, the IL-6 levels were comparable in the sera of Ctrl-11c and CKO-11c mice (Figure 6E). Therefore, Blimp-1-mediated pathway is critical for the antiviral response against JEV infection.

### Blimp-1 Regulates IRF7 Activation

**Figure 7:**
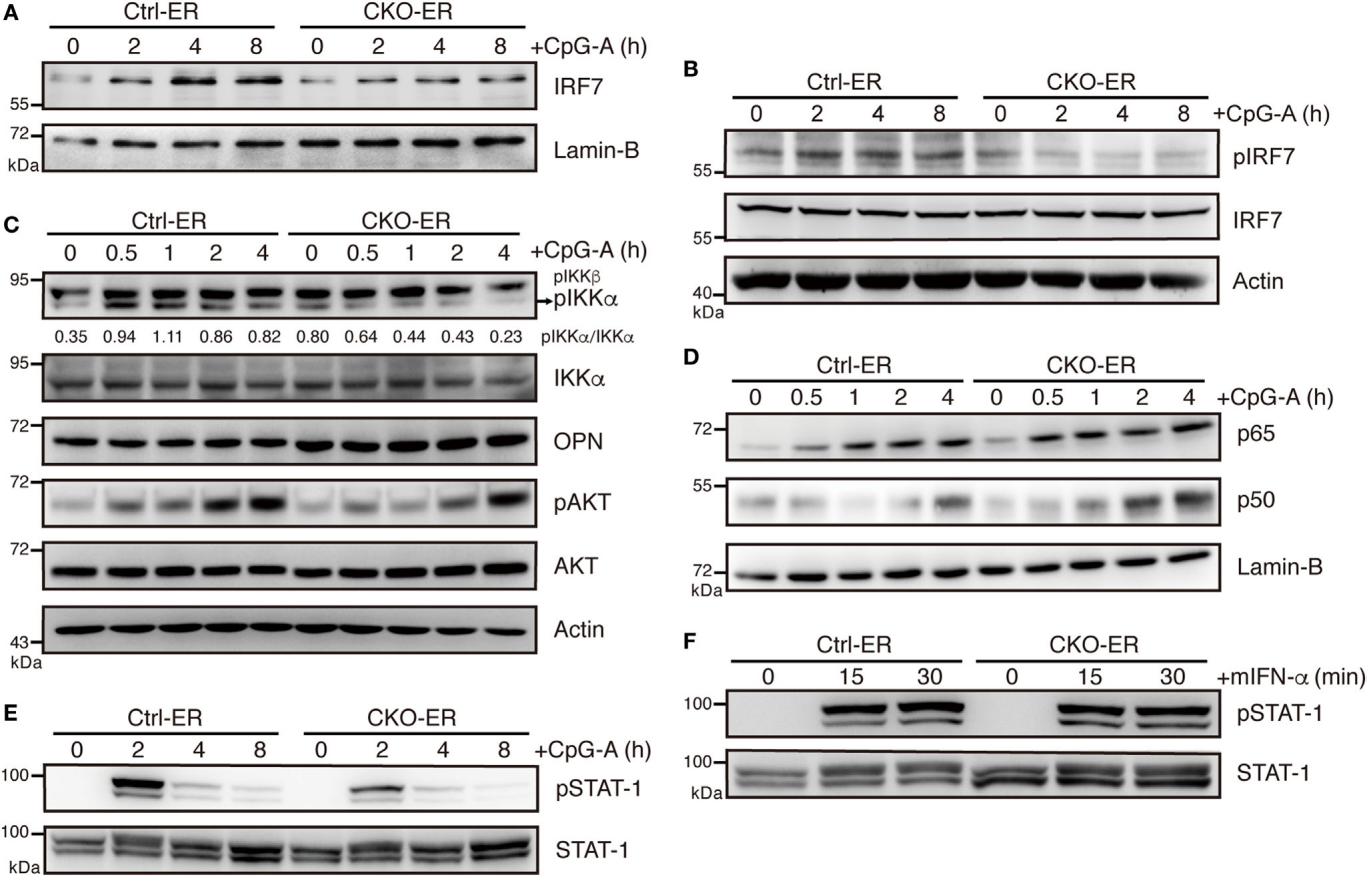
Impaired IKKα and IRF7 activation in plasmacytoid dendritic cells (pDCs) with reduced Blimp-1 expression. **(A)** Immunoblot analysis of nuclear extracts showing the levels of IRF7 in 4-OHT treated Ctrl-ER and CKO-ER FLpDCs following stimulation with 1 µM CpG-A at indicated time points. **(B)** Immunoblot analysis of total cell lysates showing the levels of IRF7 phosphorylation at Ser471/472 in 1 µM CpG-A stimulated Ctrl-ER and CKO-ER FLpDCs. **(C)** Immunoblot analysis showing the levels of phospho-IKKα at Ser176/180, OPN and phospho-AKT at Ser473, in 4-OHT treated and CpG-A stimulated Ctrl-ER and CKO-ER FLpDCs. The quantification of pIKKα was presented by the ratios of pIKKα band intensity vs. IKKα band intensity at each time point. **(D)** Immunoblot analysis using nuclear extracts of 4-OHT treated Ctrl-ER and CKO-ER FLpDCs showing the levels of p50 and p65 translocation after 1 µM CpG-A treatment at indicated time points. **(E)** Immunoblot analysis showing the levels of phosphorylated STAT1 at Tyr701 at the indicated time points in Ctrl-ER and CKO-ER FLpDCs treated with 4-OHT and then stimulated with 1 µM CpG-A at the indicated time points. **(F)** Immunoblot analysis showing levels of phosphorylated STAT1 at Tyr701 in FLpDCs in the presence of 500 U/ml mIFN-α.

Given that pDCs with reduced Blimp-1 expression had impaired IFN-I production, we determined whether the TLR-mediated signaling pathway is affected by reduced Blimp-1. Endosomal TLR7 and TLR9 are abundantly expressed in pDCs, with MyD88 serving as a mediator to provoke downstream kinase cascades and IRF7 activation. The phosphorylation and translocation of IRF7 are essential for IFN-I production in pDCs. *Irf7* KO pDCs exhibit dramatically reduced IFN-I production, but do not have reductions in other proinflammatory cytokines (33). We found that nuclear levels of IRF7 following CpG-A stimulation were greatly reduced in 4-OHT-treated FLpDCs derived from CKO-ER mice (Figure 7A). This reduction may be caused by defective phosphorylation of IRF7 at Ser437/438 (Figure 7B), because phosphorylation is required for IRF7 activation (34). Moreover, IKKα, osteopontin (OPN), and PI3K selectively regulate IFN-I production in pDCs by promoting the phosphorylation of IRF7 (35–37). IKKα activation was decreased following CpG-A treatment in Blimp-1-deficient pDCs (Figure 7C); however, OPN and the activation of PI3K downstream factor, AKT, were comparable irrespective of the presence of Blimp-1. Unlike IRF7, canonical NF-κB, p65, and p50 were activated normally by CpG-A stimulation in 4-OHT treated FLpDCs derived from CKO-ER mice (Figure 7D). This result is consistent with our notion that cytokine production is not generally affected in stimulated pDCs in the absence of Blimp-1. The IFN-I produced in the early phase response to TLR ligands amplified a positive feedback loop that signals through interferon α/β receptor (IFNAR) to activate the JAK–STAT pathway (38). To determine whether impaired IFN-I production in Blimp-1-deficient pDCs was caused by perturbed IFNAR signaling, we examined the activation of STAT1 after CpG-A stimulation in 4-OHT treated CKO-ER and Ctrl-ER FLpDCs. STAT1 phosphorylation at Tyr701 was reduced in Blimp-1-deficient FLpDCs (Figure 7E), signifying a reduction in STAT1 activation. However, this effect was attributed to perturbed production of IFN-I by Blimp-1-deficient pDCs, because comparable levels of phosphorylated STAT1 were detected in the control and Blimp-1-deficient FLpDCs after culture supplementation with mouse IFN-α (Figure 7F).

### Blimp-1 Inhibits IRAK-M Expression in pDCs

**Figure 8:**
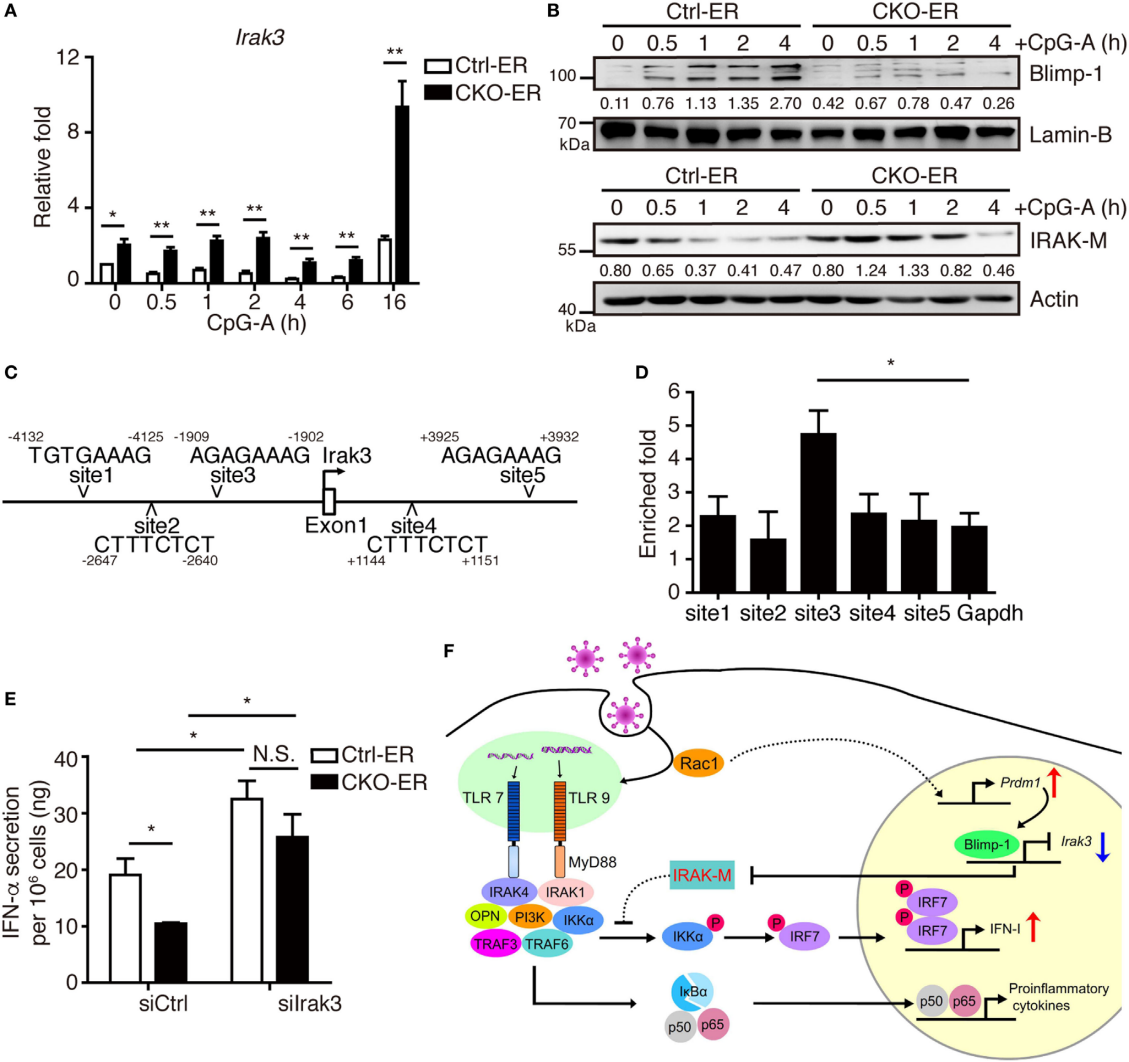
Increased IRAK-M expression in pDCs lacking Blimp-1 contributes to impaired IFN-I production. **(A,B)** RT-qPCR showing *Irak3* mRNA levels **(A)**, IRAK-M and Blimp-1 protein levels **(B)** in Ctrl-ER and CKO-ER FLpDCs treated with 4-OHT and then stimulated with 1 µM CpG-A. The quantitation of Blimp-1 in **(B)** was presented by the ratios of Blimp-1 band intensity vs. Lamin-B band intensity at each time point. The quantitation of IRAK-M in **(B)** was presented by the ratios of IRAK-M band intensity vs. actin band intensity at each time point. **(C)** Five putative Blimp-1 consensus binding sites were identified within 5 kb upstream and downstream of the *Irak3* transcriptional start site (TSS, indicated by an arrow). **(D)** ChIP assay using chromatin isolated from FLpDCs following 4 h stimulation with 1 µM CpG-A showing the levels of binding of Blimp-1 at various putative sites. *Gapdh* was used as the negative control locus. **(E)** IFN-α production by Blimp-1-deficient and control FLpDCs transfected with control siRNA (siCtrl) or siRNA-pools with three different siRNAs against *Irak3* (siIrak3) and stimulated with 1 µM CpG-A for 16 h. **(F)** Model of the action of Blimp-1 in the regulation of induction of IFN-I signaling in pDCs. Abbreviations: Rac, Ras-related C3 botulinum toxin substrate; IRAK-M, interleukin-1 receptor-associated kinase M; OPN, osteopontin; pDCs, plasmacytoid dendritic cells; IFN-I, type I IFN; siRNA, small-interfering RNA; *Irak3, interleukin-1 receptor-associated kinase 3*; ChIP, chromatin immunoprecipitation; RT-qPCR, RT-quantitative PCR. Data represent the mean ± SEM and were analyzed by two-tailed unpaired Student’s *t*-test [*n* = 4 in **(A)** and 3 in **(D,E)**]. *p < 0.05; **p < 0.01. N.S. = no significant difference.

Blimp-1 was first identified as suppressing IFN-β expression after virus infection in a human bone osteosarcoma cell line, MG63 (29). Unexpectedly, our results demonstrated that Blimp-1 positively regulates IFN-I production following virus infection in pDCs, suggesting a cell type-specific effect. To confirm this, similar levels of IFN-β production were detected in the splenic cDCs isolated from CKO-11c and Ctrl-11c mice following stimulation with the TLR3 ligand poly(I:C) or the TLR4 ligand LPS (Figures S5A,B in Supplementary Material). IRAK-M is induced in macrophages after LPS stimulation and acts as a negative regulator of TLR signaling by preventing the activation of IRAK-4/IRAK-1 (39). We tested if inhibition of IRAK-M may be required to activate the TLR-mediated signaling cascade in pDCs efficiently and whether Blimp-1 is involved in this regulation. We found that Blimp-1-deficient FLpDCs had increased IRAK-M mRNA and protein expression following CpG-A stimulation, in contrast with the downregulation of IRAK-M in stimulated Ctrl-ER FLpDCs (Figures 8A,B). According to previously identified Blimp-1 consensus binding sequences (40, 41), five putative Blimp-1 binding sites were identified within 5 kb upstream and downstream of the *Irak3* transcriptional start site (TSS, Figure 8C). Chromatin isolated from CpG-A stimulated FLpDCs was used to perform a ChIP assay to verify its binding by Blimp-1. A significant binding was present at site 3 located 1,909 bp upstream of the *Irak3* TSS (Figure 8D).

To determine whether increased IRAK-M expression contributes to the defective IFN-I production in Blimp-1-deficient pDCs, we used siRNAs to knockdown IRAK-M expression. 4-OHT treated CKO-ER and Ctrl-ER FLpDCs were transfected with siRNA-pools containing three different siRNAs specific to *Irak3* and stimulated with CpG-A (Figure S6A in Supplementary Material). IFN-α production was elevated after the knockdown of *Irak3* in CpG-A stimulated Ctrl-ER FLpDCs (Figure 8E). Notably, the knockdown of *Irak3* effectively restored the production of IFN-α in stimulated CKO-ER FLpDCs (Figure 8E), in contrast to the reduced production of IFN-α observed when these cells are transfected with control siRNA (siCtrl). The effect of siIrak3-pools on IFN-α production was specific because the knockdown efficiency of each individual siIrak3 was correlated with their effect on the restoration of IFN-α production in CKO-ER FLpDCs (Figures S6B,C in Supplementary Material). IRAK-M inhibited the production of proinflammatory cytokines, including IL-12p40, TNF-α, and IL-6, in stimulated macrophages (39). However, unexpectedly, our results suggest that IRAK-M depletion in pDCs did not influence the production of IL-6 and TNF-α (Figures S6D,E in Supplementary Material). Collectively, our results show that Blimp-1-dependent suppression of *Irak3* may accelerate IFN-I production, but not affect cytokine production, in pDCs.

## Discussion

Virus infection and stimulation by various pattern recognition receptors stimulation may trigger the expression of Blimp-1 (10, 29). Blimp-1 was originally reported to be a transcription repressor that binds to PRDI element of *IFN-β* gene promoter and inhibits sustained IFN-β expression after Sendai virus infection in human bone osteosarcoma cell lines (29). In macrophages, Blimp-1 was also reported to directly suppress the expression of murine chemokine (C-C motif) ligand 8 (*CCL8*) that modulates host defense against bacterial pathogens (42). Here, we showed that Blimp-1 was induced in pDCs, the professional IFN-I producing cells that limit viral infection, after TLR7 and TLR9 stimulation. However, to our surprise, we found that Blimp-1 did not inhibit IFN-β production; instead, Blimp-1 promotes IFN-I production and antiviral defense in pDCs. The pathway involved in the induction of Blimp-1 is unique in pDCs, which may not crucially depend on the conjugation of TLRs and ligands as suggested by our contrasting results from the kinetics of induction of Blimp-1 in TLR7- and TLR9-deficient pDCs. We show here that Rac is important for the induction of Blimp-1 in pDCs.

Rac1 is a small G protein that belongs to the Rho GTPase family, which controls many cellular events such as actin reorganization (43). Rac1 is activated by DOCK2 and acts upstream of TLR7 and TLR9 to produce IFN-I in pDCs. Rac is required for the non-specific endocytosis, macropinocytosis, in splenic DCs (44), and the endocytic capacity of DCs is enhanced after stimulation with TLR ligands (24). The activation of Rac1 has also been implicated in virus infection. Studies showed that Rac1 is involved in the suppression of H1N1 virus replication (45), and that activation of Rac1 after HSV-1 infection downregulates virus infectivity (46). Furthermore, Rac activation promotes caveolin-mediated JEV internalization (47). We suspect that Blimp-1 activation after virus infection in pDCs is also Rac-dependent. Using a potent Rac inhibitor, EHop-016, which inhibits Rac activity by targeting to the GEF binding pocket of Rac (26), we demonstrated that Blimp-1 induction in response to the stimulation with TLR7 and TLR9 ligands depends on the activation of Rac. Although, our and others’ data (25) indicate that Rac1 is activated after stimulation with TLR7 and TLR9 ligands in pDCs, we cannot rule out the possibility that other Rac family members may also involve as EHop-016 inhibits all Rac family members. Although we found that the induction of Blimp-1 in pDCs is mediated through Rac-1, we here do not know the exact mechanisms causing the defective Rac-1 activation in *Tlr7* KO pDCs. Studies have just begun to reveal that the regulation of these two endosomal TLRs, TLR7 and TLR9, may be quite different. For example, TLR9, but not TLR7, needs UNC93B1, a multipass transmembrane protein, to traffic from plasma membrane to the endosome (48). TLR9 requires UNC93B1-mediated recruitment of AP-2 to ship into endolysosomes, while TLR7 utilizes alternative trafficking pathways. In terms of their functions, in lupus-prone mice, TLR7 and TLR9 have opposing roles in inflammation: TLR9 is required for inflammatory regulation but TLR7 promotes lymphocytes activation and serum IgG production (49). Therefore, it is possible that TLR7, but not TLR9, employs a feedback upregulation for Rac-1 activation.

We here find that the development of pDCs is not affected by Blimp-1 because the absolute pDC numbers in the spleen, the mRNA levels of various key transcription factors, and the expression of pDC markers were not altered by the deletion of *Prdm1*. Furthermore, the activation of pDCs following stimulation with TLR9 ligands was not influenced by the absence of Blimp-1. This is in contrast to our previous findings showing the role of Blimp-1 in cDCs where Blimp-1 deficiency led to the impaired up-activation of MHCII and other activation markers after TNF-α and stimulation with various TLR ligands in BM-derived DCs (8), showing the cell type-specific action of Blimp-1. We suspect that in pDCs, Blimp-1 may participate in the regulation of TLR downstream signaling independent to the activation of pDCs. Endosomal TLR7 and TLR9 are highly expressed in pDCs compared with other splenic DC subsets (50). Upon stimulation, TLR7 and TLR9 undergo conformational changes and recruit downstream factors to form the cytoplasmic transductional translational processor that transduces signals through phosphorylation and unbiquitination (51), finally activating IRF7 for robust IFN-I production. The expression of IRF7 was also controlled by NFATC3 in pDCs (52). In Blimp-1-deficient pDCs, impaired IRF7 phosphorylation and nuclear translocation was found; however, NF-κB activation and proinflammatory cytokine production were not affected. IKKα, osteopontin, and PI3K are necessary for IFN-I production, but not the secretion of other proinflammatory cytokines, by promoting the activation of IRF7 in pDCs (35–37). Our findings that Blimp-1 affects IKKα, IRF7 activation, and IFN-I production support these previous reports. IFN-I produced in response to TLR ligands in the early phase amplifies a positive feedback loop that signals through activation of JAK–STAT pathway *via* IFNAR (38). Our finding that impaired STAT-1 activation in Blimp-1-deficient pDCs was restored by supplemental IFN-α excludes the idea that Blimp-1 acts downstream of IFNAR signaling in IFN responses. Our data demonstrated the importance and the action of Blimp-1 in the sequential pathways of IFN-I production in pDCs. Furthermore, the function of Blimp-1 in the regulation of IFN-I in pDCs is cell type specific.

Interleukin-1 receptor-associated kinase M is a negative regulator of TLR signaling (39), but its expression kinetics appears to differ among cell types. Low expression of IRAK-M was reported in macrophages in the steady state, and both mRNA and protein levels were increased at 6–24 h after LPS stimulation (39). Furthermore, in *Irak3* KO macrophages, increased IL-12p40, TNF-α, and IL-6 production was observed after stimulation with various pathogen-associated molecular patterns (39). However, in human pDCs stimulated with R837, the high levels of IRAK-M declined rapidly. Moreover, knockdown of IRAK-M in human pDCs increased IFN-I production after TLR7 stimulation (19). We found that IRAK-M mRNA and protein levels were increased in TLR9-stimulated Blimp-1-deficient FLpDCs. According to our ChIP data, Blimp-1 directly bound to the promoter region of *Irak3* at 1,909 bp upstream of the TSS, suggesting that Blimp-1 may directly suppress the expression of IRAK-M. Therefore, the Blimp-1-mediated suppression of *Irak3* might be important for the regulation of IFN-I production in pDCs because impaired IFN-I production was restored after knockdown of *Irak3* in Blimp-1 deficient FLpDCs in response to TLR9 simulation.

Our findings regarding the role of Blimp-1 in the regulation of IFN-I production in pDCs may have clinical relevance, such as in viral infection. IFN-I is one of the most important mediators against viral infection (53). Mosquito-borne JEV belongs to the Flaviviridae family, which causes up to 70,000 viral encephalitis cases annually (54). Previous studies have demonstrated the necessity of IFN-I in JEV infection both *in vitro* and *in vivo* (15, 31). We here also showed that anti-PDCA-1 administration significantly accelerates the death of JEV infected mice. Administration of anti-PDCA-1 antibody may affect other non-pDC cell types, particularly after viral infection (55). To avoid the inadvertent effects on deleting other immune cells, we stopped the anti-PDCA-1 antibody administration after JEV infection. With this approach, we found that the frequency and cell numbers of other cell lineages, including cDCs, myeloid cells, B cells, T cells, NK cells, and NKT cells, remained unchanged between anti-PDCA-1 antibody and control antibody treated groups before and 3 days after JEV infection. Therefore, our results indicated the importance of pDC-induced IFN-I production for defense against JEV infection. More importantly, decreased IFN-α production in serum and elevated virus replication in the brain were observed in CKO-11c mice after JEV infection. Furthermore, similar to the effect of Blimp-1 on the production of proinflammatory cytokines after stimulation with TLR ligands in pDCs, there were no differences in the cytokine production after JEV infection in CKO-11c mice. A negative role of TRIM29 in DNA virus infection in DCs through inhibiting the expression of stimulator of interferon genes, a key molecule in cytosolic DNA-sensing pathway, has been reported (56). Our results showed that Blimp-1 is important for the production of IFN-I in pDCs after CpG treatment and HSV-1 infection. It will be interesting to determine the role of Blimp-1 in DNA virus infection *in vivo*.

In conclusion, we demonstrated a Rac-mediated pathway is involved in the induction of Blimp-1 following the exposure of pDCs to TLR ligands. Blimp-1 suppresses *Irak3*, which efficiently relieves the negative regulation of TLR signaling and allows increased IFN-I production (Figure 8F). The Rac/Blimp-1/IRAK-M/IFN-I pathway identified in this study may be a new target pathway to selectively modulate the levels of IFN-I, but not cytokines, for the control of antiviral responses.

## Ethics Statement

Animal experimental protocols were approved by IACUC of Academia Sinica. The consent procedures of collection of samples from healthy donors were approved by the Academia Sinica Research Ethics Committee.

## Author Contributions

K-IL conceived and designed the study. Y-AK, Y-HC and J-JL performed the experiments. Y-AK, Y-HC, J-JL and K-IL analyzed the data. C-HL, T-HC, Y-PH and Y-LL provided crucial animals and reagents. Y-AK. and K-IL wrote the manuscript.

## Conflict of Interest Statement

The authors declare that the research was conducted in the absence of any commercial or financial relationships that could be construed as a potential conflict of interest.
