# Supplementary material for: Blimp-1-Mediated Pathway Promotes Type I IFN Production in Plasmacytoid Dendritic Cells by Targeting to Interleukin-1 Receptor-Associated Kinase M
Source: Front Immunol. 2018 Aug 7;9:1828. doi: 10.3389/fimmu.2018.01828 (PMC6091234; doi:10.3389/fimmu.2018.01828)
Supplement: Supplementary file 1 [file presentation_1.pdf]

*Supplementary Material*

# Blimp-1-Mediated Pathway Promotes Type I IFN Production in Plasmacytoid Dendritic Cells by Targeting to Interleukin-1 Receptor-Associated Kinase M

Yi-An Ko[1,2], Yueh-Hsuan Chan[1], Chin-Hsiu Liu[1,3], Jian-Jong Liang[4], Tsung-Hsien Chuang[5], Yi-Ping Hsueh[6], Yi-Ling Lin[4], and Kuo-I Lin[1]*

[1]Genomics Research Center, Academia Sinica, Taipei, Taiwan; [2]Institute of Microbiology and Immunology, National Yang-Ming University, Taipei, Taiwan; [3]Ph.D. Program in Translational Medicine, Kaohsiung Medical University and Academia Sinica, Division of Allergy, Immunology and Rheumatology, Taipei Tzu Chi Hospital, Buddhist Tzu Chi Medical Foundation, New Taipei City, Taiwan; [4]Institute of Biomedical Sciences, Academia Sinica, Taipei, Taiwan; [5]Immunology Research Center, National Health Research Institutes, Miaoli, Taiwan; [6]Institute of Molecular Biology, Academia Sinica, Taipei, Taiwan.

*Corresponding author:
Kuo-I Lin (kuoilin@gate.sinica.edu.tw)

**Supplementary Figures**

**Supplementary Figure S1. pDC gating strategy**
**Supplementary Figure S2. Blimp-1 deletion efficiency in DCs from the CKO-11c mice.**
**Supplementary Figure S3. pDC development was not affected in Blimp-1-deficient FLpDCs.**
**Supplementary Figure S4. pDCs were important for protection of mice from JEV infection.**
**Supplementary Figure S5. Blimp-1 was dispensable for IFN-I production in cDCs.**
**Supplementary Figure S6. Knockdown of Irak3 restored IFN-I production in Blimp-1 deficient pDCs.**

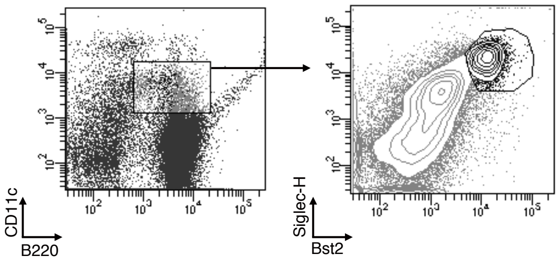


**Supplementary Figure S1. pDC gating strategy.** The single cell suspensions from mouse spleen were obtained for gating pDCs, identified as CD11c$^{int}$B220$^{+}$ Siglec-H$^{+}$Bst2$^{+}$.

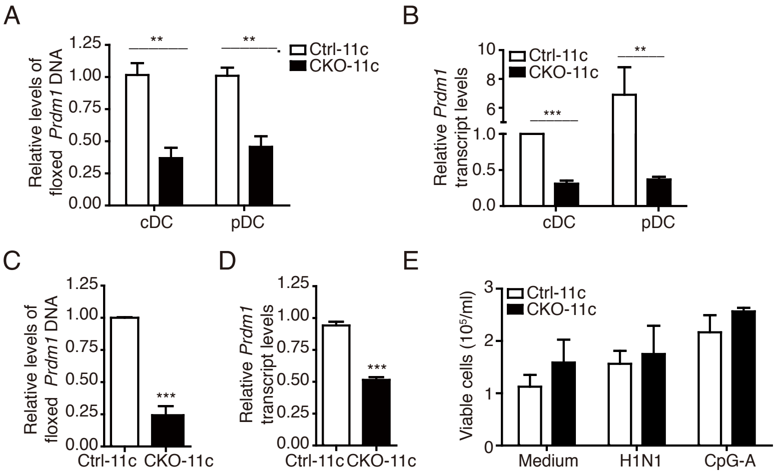


**Supplementary Figure S2. Blimp-1 deletion efficiency in DCs from the CKO-11c mice.** **(A, B)** qPCR using genomic DNA **(A)** and RT-qPCR using cDNA **(B)** confirming the deletion of *Prdm1* in CKO-11c cDCs and pDCs. Splenic pDCs and cDCs were isolated and sorted from Ctrl-11c or CKO-11c mice. **(C, D)** qPCR using genomic DNA **(C)** and RT-qPCR using cDNA **(D)** showing the deletion of *Prdm1* in CD11c$^{+}$ DCs isolated from the BM of Ctrl-11c or CKO-11c mice. **(E)** The viable pDC counts in Figures 3D−F. Results represent the mean ± SEM and were analyzed by two-tailed unpaired Student's *t* test (n=4 in A, 2−4 in B, 5−7 in C, 5 in D and 3−4 in E). $^{**}p < 0.01$; $^{***}p < 0.001$.

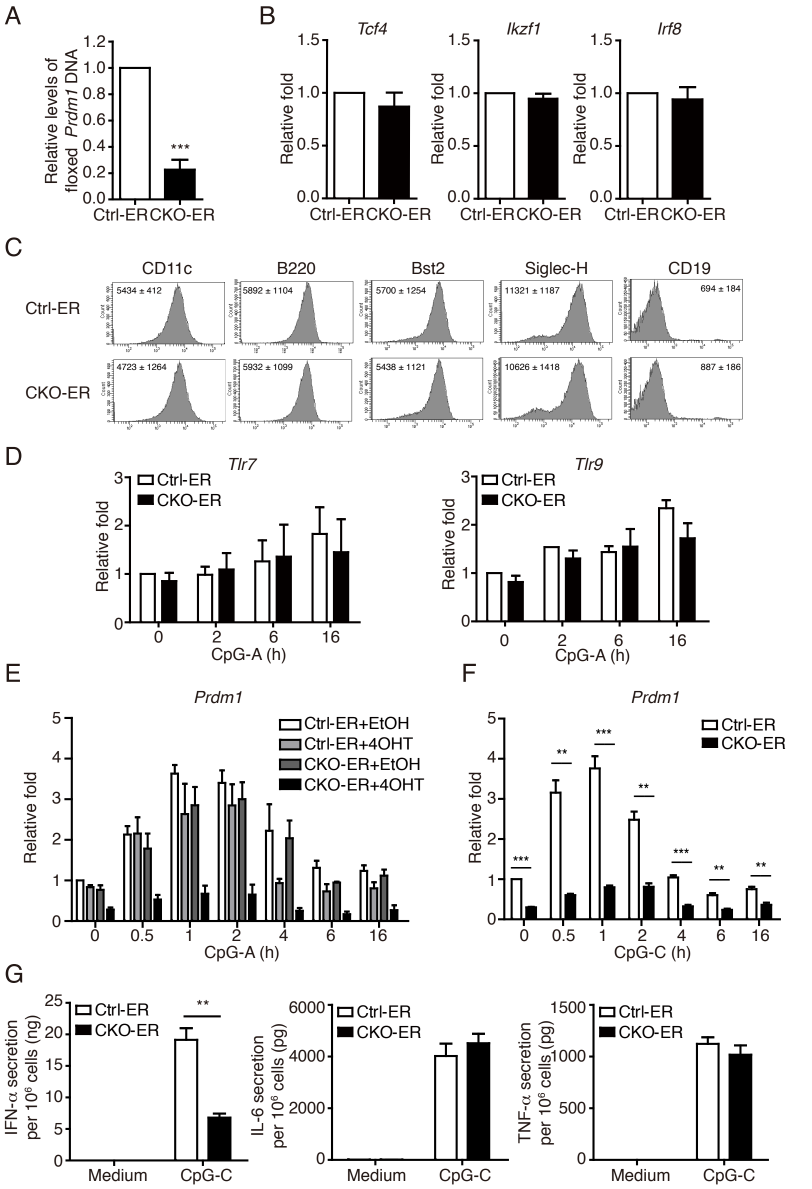


**Supplementary Figure S3. pDC development was not affected in Blimp-1-deficient FLpDCs. (A)** qPCR using genomic DNA from 4-OHT treated Ctrl-ER and CKO-ER FLpDCs showing a significant deletion of *Prdm1* at day 9. **(B)** RT-qPCR showing the mRNA levels of E2-2 (*Tcf4*), Ikaros (*Ikzf1*) and IRF8 in Ctrl-ER and CKO-ER FLpDCs at day 9. **(C)** Flow cytometric analysis of pDC maturation markers on Ctrl-ER and CKO-ER FLpDCs. The MFI of each histogram is indicated. **(D)** RT-qPCR showing *Tlr7* and *Tlr9* mRNA levels at indicated time points in 4-OHT treated Ctrl-ER and CKO-ER FLpDCs following 1 μM CpG-A stimulation. **(E)** RT-qPCR showing the mRNA levels of Blimp-1 in EtOH or 500 nM 4-OHT treated Ctrl-ER and CKO-ER FLpDCs after 1 μM CpG-A stimulation at various time points. **(F)** RT-qPCR showing Blimp-1 mRNA levels in FLpDCs from Ctrl-ER and CKO-ER mice treated with 500 nM 4-OHT and then stimulated with 1 μM CpG-C at indicated time points. **(G)** 4-OHT treated FLpDCs cultured from Ctrl-ER and CKO-ER mice were stimulated with 1 μM CpG-C or medium alone for 16 hrs, followed by ELISA to measure the levels of IFN-α, IL-6 and TNF-α production. Results represent the mean ± SEM and were analyzed by two-tailed unpaired Student's *t* test (n=4 in A and B, 3−4 in C, 2 in D and 3 in E−G). $^{**}p < 0.01$; $^{***}p < 0.001$.

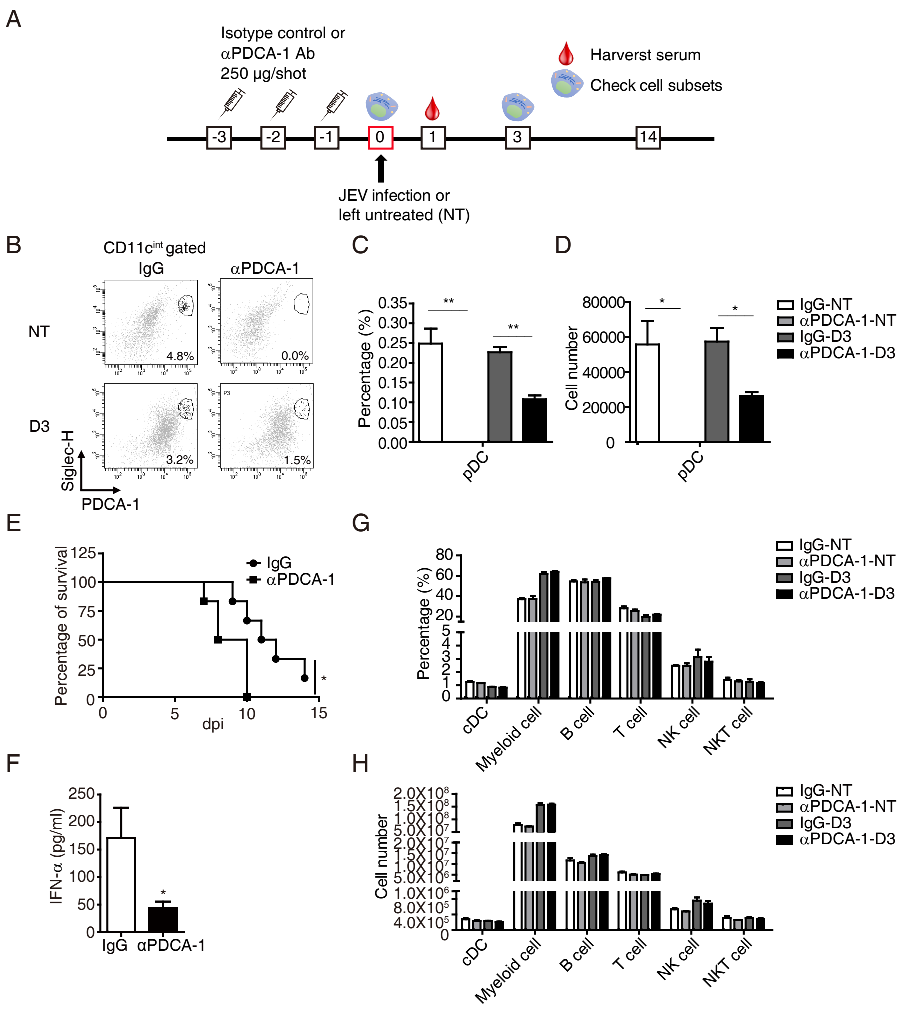


**Supplementary Figure S4. pDCs were important for protection of mice from JEV infection. (A)** Experimental scheme of pDC depletion and JEV infection. pDCs in C57BL/6 mice were depleted by i.p. injection of anti-PDCA-1 antibody for three times (250 μg/injection), or injected with control rat IgG, at 24-hr intervals. Mice were infected with JEV 24 hrs after the third antibody injection or left untreated (NT). Various immune cell subsets in spleen were examined at D0 (NT) and three days after infection (D3). **(B–D)** Flow cytometric analysis showing a significant reduction of the frequency of CD11c$^{int}$Siglec-H$^{+}$PDCA-1$^{+}$ pDCs in spleen after anti-PDCA-1 antibody injection, as compared with that in control antibody injection, in NT and JEV infected groups **(B)**. The percentage and total cell number of splenic pDCs were shown in **(C)** and **(D)**, respectively. **(E)** Survival rates of C57BL/6 mice injected with control IgG or anti-PDCA-1 antibody after JEV infection. **(F)** ELISA showing serum IFN-α levels in control IgG and anti-PDCA-1 antibody treated mice 24 hrs after JEV infection. **(G, H)** The percentage **(G)** and total cell number **(H)** of various immune cell subsets, including cDC (CD11c$^{high}$MHCII$^{high}$CD49b$^{-}$), myeloid cell (CD11b$^{+}$), B cell (CD19$^{+}$), T cell (CD3$^{+}$CD49b$^{-}$), NK cell (CD3$^{-}$CD49b$^{+}$) and NKT cell (CD3$^{+}$CD49b$^{+}$), were detected in control antibody or anti-PDCA-1 antibody injected mice without or with JEV infection for 3 days. Data represent the mean ± SEM and were analyzed by two-tailed unpaired Student's *t* test (n=3 in C and D, 6 in F and 3 in G and H). Data in **(E)** were analyzed by log-rank (Mantel-Cox) test (n=6). $*p < 0.05$; $**p < 0.01$.

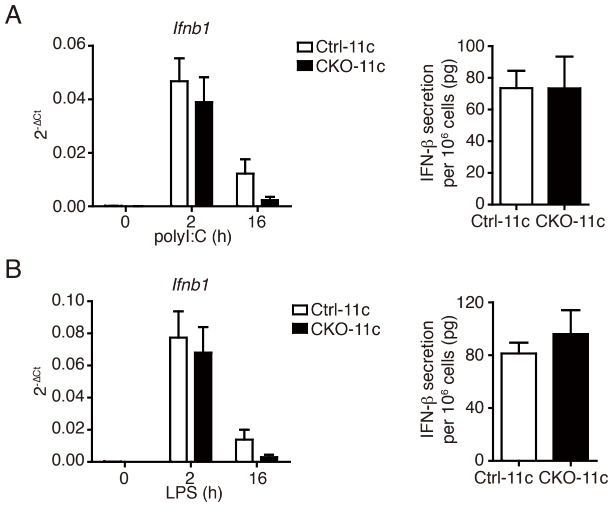


**Supplementary Figure S5. Blimp-1 was dispensable for IFN-I production in cDCs. (A, B)** RT-qPCR (left panel) and ELISA (right panel) showing the levels of IFN-β in splenic cDCs isolated from Ctrl-11c and CKO-11c mice after 50 ng/ml polyI:C **(A)** and 10 ng/ml LPS **(B)** stimulation. Results represent the mean ± SEM and were analyzed by two-tailed unpaired Student's *t* test (n=3−4 in A and B).

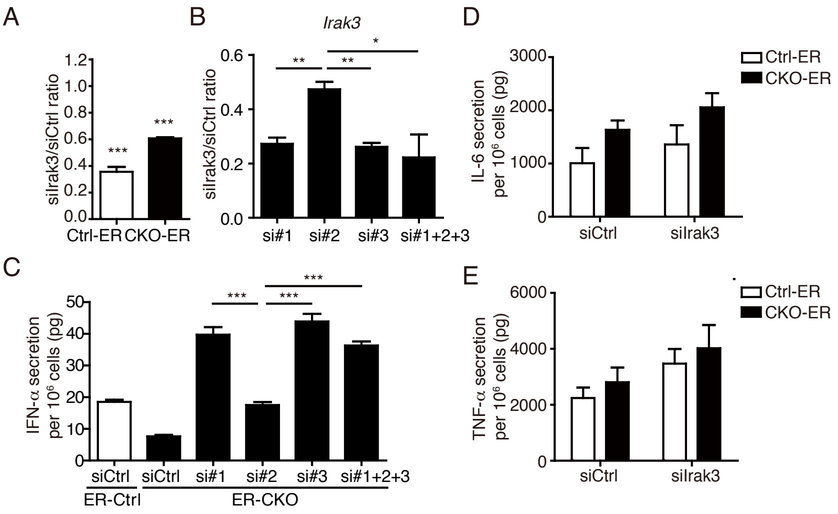


**Supplementary Figure S6. Knockdown of Irak3 restored IFN-I production in Blimp-1 deficient pDCs. (A)** siIrak3-pools knockdown efficiency in Ctrl-ER and CKO-ER FLpDCs treated with 4-OHT compared with those of siCtrl-transfected groups. **(B)** RT-qPCR showing the *Irak3* mRNA levels in 4-OHT treated CKO-ER FLpDCs transfected with three different siRNAs against *Irak3* (si#1, si#2 and si#3) or *Irak3* siRNA-pools (si#1+2+3). **(C)** IFN-α production by Blimp-1-deficient and control FLpDCs transfected with siCtrl, siIrak3 (si#1, si#2 or si#3) or siIrak3-pools (si#1+2+3) and stimulated with 1 μM CpG-A for 16 hrs. **(D, E)** ELISA showing the levels of IL-6 **(D)** and TNF-α **(E)** production in Blimp-1 sufficient (Ctrl-ER) and deficient (CKO-ER) FLpDCs transfected with siCtrl or siIrak3-pools and stimulated with 1 μM CpG-A for 16 hrs. Results represent the mean ± SEM and were analyzed by two-tailed unpaired Student's $t$ test (n=3 in A–E). *$p < 0.05$; **$p < 0.01$. ***$p < 0.001$.
